# Horizontal Eye Position in Thyroid Eye Disease: A Retrospective Comparison with Normal Individuals and Changes after Orbital Decompression Surgery

**DOI:** 10.1371/journal.pone.0114220

**Published:** 2014-12-03

**Authors:** Yasuhiro Takahashi, Hirohiko Kakizaki

**Affiliations:** Department of Ophthalmology, Aichi Medical University, Nagakute, Aichi, Japan; Sun Yat-sen University, China

## Abstract

**Objective:**

To compare horizontal eye positions between proptotic thyroid eye disease patients and normal individuals, and to examine positional changes after orbital decompression surgery in thyroid eye disease patients.

**Methods:**

The present case-controlled and retrospective comparative study included 78 proptotic thyroid eye disease patients who underwent bilateral orbital decompression surgery [lateral orbital wall decompression (Group L), 47 patients; medial orbital wall decompression (Group M), 9 patients; and balanced orbital decompression (Group B), 22 patients] and 143 age-matched healthy volunteers as controls. The interpupillary distance was measured to determine horizontal eye positions before and 3 months after surgery in thyroid eye disease patients and was also examined in control eyes. Horizontal eye shifts were calculated by subtracting postoperative from preoperative interpupillary distances.

**Results:**

Preoperative interpupillary distances in thyroid eye disease patients were significantly larger than in controls. The interpupillary distances were significantly decreased postoperatively in Groups M and B, but were significantly increased in Group L. The order of the magnitude of the horizontal shifts was Groups M>B>L.

**Conclusions:**

Proptotic thyroid eye disease patients preoperatively showed laterally displaced eyes in comparison with controls. However, the eyes shifted medially after the medial orbital wall decompression and the balanced orbital decompression, although the former showed more shift. Medial orbital wall or balanced orbital decompression can be used to correct both lateral and anterior displacement of the eyes.

## Introduction

Eye position is a key aesthetic element [1]. Lateral (hypertelorism) and anterior displacement of the eye (proptosis) are representative eye malpositions that disfigure patients' faces. Horizontal eye positions correlate with anteroposterior positions in the normal population; i.e., a more lateral eye position implies a more anterior eye position [2], [3], [4].

Thyroid eye disease (TED) is one of the most representative entities that cause proptosis. Increased orbital fat and enlarged extraocular muscles occupy the limited orbital space, pushing the eye anteriorly [5]. However, whether an eye is displaced laterally in proptotic TED patients has not been examined.

Orbital decompression surgery is commonly used for correcting proptosis in TED patients. However, whether horizontal eye shifts occur after orbital decompression surgery is still unclear. Although two reports have indicated horizontal shifts occurring after orbital decompression surgery, the results were contradictory [6], [7]. Alsuhaibani *et al*. showed that the eyes became closer in the horizontal plane, by 2.6 mm on average, after balanced orbital decompression surgery (lateral orbital wall plus medial orbital wall decompressions) [6]. However, Fichter *et al*. reported that the interpupillary distance increased by 1.5 mm, on average, after *en bloc* resection of the lateral orbital wall, including the whole lateral orbital rim [7].

We therefore examined differences in horizontal eye positions between proptotic TED patients and normal individuals, and also examined changes in the horizontal eye position after orbital decompression surgery.

## Materials and Methods

### Patients and Measurements

In the present case-controlled and retrospective comparative study, we reviewed all proptotic TED patients who underwent orbital decompression surgery, performed by one oculoplastic surgeon (HK), during August 2006 through February 2014. Among them, we included all patients who underwent lateral orbital wall decompression, balanced orbital decompression, or medial orbital wall decompression, bilaterally, for ease of comparison among surgical procedures. Patients with a follow-up period of less than 3 months, or with lack of clinical data, were excluded from this study. As controls, we included age-matched volunteers without eyelid lag, eyelid retraction, high myopia (> 6 diopters) [4], or a past history of any orbital disease. We obtained Institutional Review Board (IRB) approval from the Ethics Committee at Aichi Medical University (No. 13–137), and followed the tenets of the Declaration of Helsinki. The IRB granted a waiver of informed consent from the patients for this study, based on the ethical guidelines for epidemiological research established by the Japanese Ministry of Education, Culture, Sports, Science and Technology, and Ministry of Health, Labour and Welfare because this study was a retrospective chart review, not an interventional study, and because it was difficult to get consent from all of the patients studied many years prior. However, the IRB requested us to present an outline of this study to the public via the Website of Aichi Medical University, to provide an opportunity for patients to refuse participation in this study. Patient records were anonymized and were de-identified prior to analysis.

Information collected from medical records of the TED patients included age, gender, and surgical procedures. The anteroposterior eye position was measured in the TED patients and in the controls, using a Hertel exophthalmometer, by one of the authors (HK). The measurements in TED patients were done preoperatively, and also 3 months postoperatively. We used average Hertel exophthalmometry values of the right and left sides to analyze the relationships between the horizontal and anteroposterior eye positions, as described in the “Statistical Analysis” section. Exophthalmometry values were, therefore, defined as follows: (right + left Hertel exophthalmometry measurement value)/2. Backward eye shift was calculated as follows: [(right preoperative − right postoperative Hertel exophthalmometry measurement value) + (left preoperative − left postoperative Hertel exophthalmometry measurement value)]/2.

The interpupillary distance was measured to determine horizontal eye positions in the TED patients and in the controls, with an autorefractor/keratometer (ARK-700A; Nidek Co., Ltd., Aichi, Japan) or an autorefractor/keratometer/tonometer (TONOREF II; Nidek Co., Ltd., Aichi, Japan). The measurements in TED patients were done preoperatively and 3 months postoperatively. The measurements, using both ARK-700A and TONOREF II, are not influenced by strabismus because the instrument tracks each eye until optimal positioning. The horizontal eye shift was calculated by subtracting postoperative from preoperative interpupillary distances. Positive values of horizontal eye shifts were defined when the distance of the eyes became closer to each other.

TED patients were subdivided into three groups according to the surgical procedure: Group L (lateral), M (medial), and B (balance) included patients who had undergone bilateral lateral orbital wall decompression, bilateral medial orbital wall decompression, and bilateral balanced decompression, respectively.

### Surgery

Surgery was performed according to the grade of proptosis [8], [9]. In patients with mild to moderate proptosis, a lateral orbital wall decompression was performed, and in those with severe proptosis, a balanced orbital decompression was selected. When patients with mild to moderate proptosis showed a small lateral orbital wall, or when patients sustained a dysthyroid optic neuropathy, the medial orbital wall decompression was performed [10]. All surgeries were performed under general anesthesia with the aid of binocular loupes (high resolution prismatic × 2.5, 340 mm/13 inches; Heine, Herrsching, Germany).

**Figure 1:**
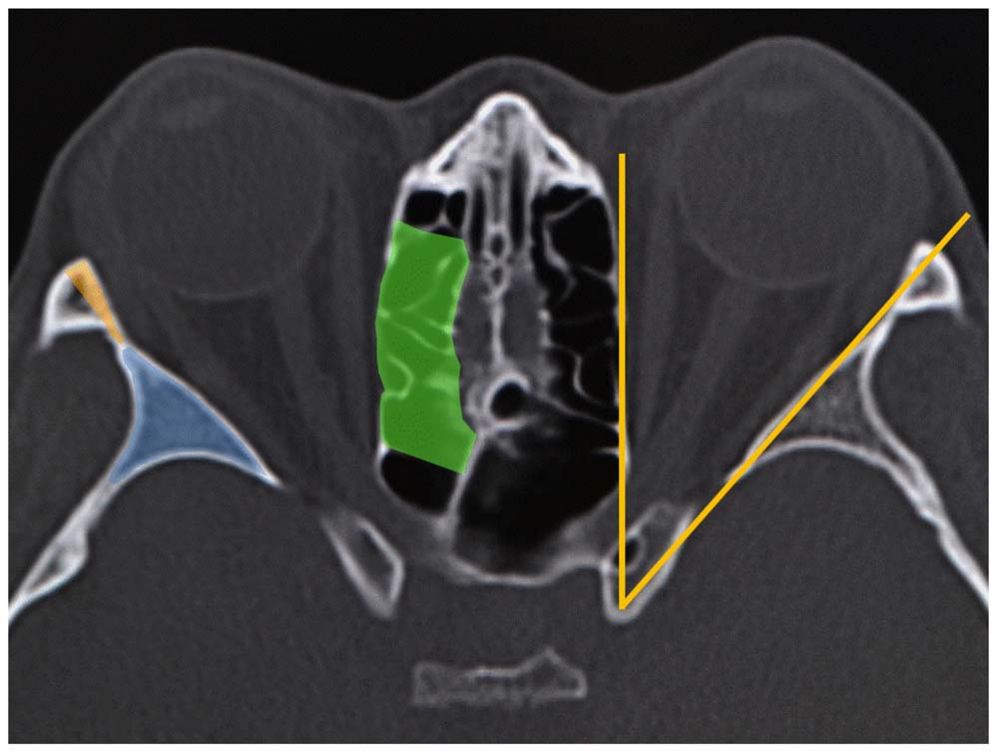
Axial computed tomographic image of the orbit. In the lateral orbital wall decompression, the greater wing of the sphenoid (the blue area), and “the medial eaves” of the lateral orbital rim (the orange area) were removed. In the medial orbital wall decompression, the medial orbital wall with its periosteum and the septa of the ethmoid air cells (the green area) were removed. The lateral walls are oriented to an angle of 45 degrees lateral to the sagittal plane, whereas the medial walls of each orbit are oriented in the sagittal plane (the yellow solid lines).

For lateral orbital wall decompression, the Berke incision (a skin incision along the lateral canthal rhytids, with lateral canthotomy and cantholysis) [11] or the swinging eyelid approach (the Berke incision approach plus an incision of the inferior conjunctival fornix) [12] was used. Although the lateral orbital wall comprises the sphenoid bone, frontal bone, zygomatic bone, and maxillary bone, the lateral orbital wall is mainly decompressed from the greater wing of the sphenoid (Fig. 1) [13]. We first removed “the medial eaves” of the lateral orbital rim to secure a large surgical field (Fig. 1). The deep lateral orbital wall was then removed, up to the cortical bone of the posterior, superior, and lateral borders of the greater wing [13]. After incising the periosteum on the lateral wall, the orbital fat was removed from the inferolateral intraconal space.

Medial orbital wall decompression was performed through a transcaruncular approach [14]. The orbital fat was first removed in some patients to secure a surgical field. The medial orbital wall, with its periosteum and the septa of the ethmoid air cells, were removed, starting at the level 10 mm posterior, to the posterior lacrimal crest [15], and extending to the level of the posterior ethmoidal foramen (Fig. 1) [13].

### Statistical Analysis

Age, interpupillary distance, exophthalmometry values, and horizontal and backward eye shifts were expressed as the mean value ± standard deviation. Age, interpupillary distance, and exophthalmometry values were compared between TED patients and controls, using the Mann–Whitney U test in male patients, and the Student's *t*-test in female patients, depending on the number of patients in each group. When the number was <20, the Mann-Whitney U test was used. We compared pre- and postoperative interpupillary distances in each group, using a paired *t*-test. Intergroup differences in age, pre- and postoperative interpupillary distances, preoperative exophthalmometry values, and the horizontal and backward eye shifts were examined using a one-way analysis of variance (ANOVA) and the Tukey–Kramer test. Ratios of sides with a positive horizontal shift were compared between the groups, using the Fisher's exact probability test or a chi-square test for independent variables, depending on the number of sides. When the sample number was less than 5, the Fisher's exact probability test was used. The relationship between horizontal and backward eye shifts was examined using the Pearson's correlation coefficient test. Age, interpupillary distances, exophthalmometry values, and horizontal and backward eye shifts were compared between genders, using the Mann–Whitney U test in each patient group, and the Student's *t*-test in controls. Statistical analysis was performed using IBM SPSS Statistics version 22 software (IBM Japan, Tokyo, Japan). A value of *P*<0.05 was considered to be statistically significant.

## Results

All data and the results of the statistical analyses are summarized in Tables 1, 2, 3, and 4. Groups L, M, and B included 47, 9, and 22 patients, respectively. This study also enrolled 143 healthy volunteers as the age-matched controls.

**Table 1:** Summary of patient data, measurement results, and statistical comparisons between preoperative measurement values in thyroid eye disease (TED) patients and measurement values in controls.

	TED patients (preoperative values)	Control	P value
Number			
Male	14	59	-
Female	64	84	-
Age (years)			
Male	37.9±9.3 (26–62)	36.3±10.1 (24–65)	0.365^a^
Female	38.1±12.4 (16–76)	36.5±10.0 (24–57)	0.382^b^
Interpupillary distance (mm)			
Male	70.4±3.5 (65.0–79.0)	64.7±3.5 (56.0–72.0)	< 0.001^a^
Female	64.7±2.8 (59.0–71.0)	61.9±3.2 (55.0–69.0)	< 0.001^b^
Exophthalmometry value (mm)			
Male	22.41±3.11 (17.25–26.50)	14.05±2.15 (10.00–19.00)	< 0.001^a^
Female	21.83±2.30 (17.00–27.50)	14.28±2.00 (9.25–19.00)	< 0.001^b^

Statistical comparison using the ^a^Mann–Whitney U test or ^b^Student's *t*-test.

**Table 2:** Summary of patient data and measurement results in each group, and statistical comparisons between pre- and postoperative values between the groups.

	Group L	Group M	Group B	*P* value: Intergroup difference			
				One-way ANOVA	L vs M	L vs B	M vs B
Number of patients	47	9	22				
Males/Females	9/38	2/7	3/19				
Age (years)	35.4±10.0 (16–62)	50.9±14.6 (31–76)	38.4±11.5 (19–60)	0.001	0.001^a^	0.547^a^	0.014^a^
Interpupillary distance (mm)							
Preoperative	65.6±3.6 (59.0–73.0)	65.6±3.0 (62.0-71.0)	66.2±4.0 (61.0–79.0)	0.815		-	-
Postoperative	66.1±3.6 (58.0–74.0)	60.2±4.6 (52.0–66.0)	64.4±4.7 (55.0–78.0)	0.001	< 0.001^a^	0.235^a^	0.029^a^
*P* value: Preoperative vs Postoperative	0.007^b^	< 0.001^b^	0.029^b^				
Horizontal eye shift (mm)	−0.5±1.3 (−3.0–3.0)	5.3±2.8 (2.0–10.0)	1.8±2.2 (-3.0–6.0)	<0.001	< 0.001^a^	< 0.001^a^	< 0.001^a^
Number of patients with positive horizontal shift	8 (17.0%)	9 (100%)	16 (72.7%)	-	< 0.001^c^	< 0.001^d^	0.101^c^
Preoperative exophthalmometry value (mm)	21.25±2.05 (17.00–25.50)	20.97±2.47 (17.25–25.00)	23.81±2.32 (19.00–27.50)	<0.001	0.935^a^	< 0.001^a^	0.004^a^
Backward eye shift (mm)	5.10±1.51 (2.25–9.25)	3.14±1.05 (1.75–5.00)	5.93±1.31 (3.00–8.25)	<0.001	0.001^a^	0.065^a^	< 0.001^a^

Statistical comparison using ^a^Tukey–Kramer test, ^b^paired *t*-test, ^c^Fisher's exact probability test, or ^d^Chi-square test for independent variables.

**Table 3:** Gender-specific measurement results and statistical comparisons in in thyroid eye disease patients.

		Number of patients	Age (years)	Interpupillary distance (mm)			Preoperative exophthalmometry value (mm)	Backward eye shift (mm)
				Preoperative	Postoperative	Horizontal eye shift		
Total	Male	14	37.9±8.9 (26–62)	70.4±3.4 (65.0–79.0)	69.5±3.8 (64.0–78.0)	0.9±2.2 (−2.0–7.0)	22.41±3.11 (17.25–26.50)	4.46±1.99 (1.75–9.25)
	Female	64	38.1±12.4 (16–76)	64.7±2.8 (59.0–71.0)	64.0±3.9 (52.0–71.0)	0.8±2.7 (−3.0–10.0)	21.84±2.30 (17.00–27.50)	5.26±1.48 (2.25–8.25)
	P value	-	0.966	< 0.001	< 0.001	0.836	0.431	0.095
Group L	Male	9	37.1±10.0 (26–62)	69.6±2.8 (65.0–73.0)	69.6±2.8 (65.0–74.0)	0±1.3 (−2.0–3.0)	21.75±2.62 (17.50–24.50)	4.86±2.06 (2.25–9.25)
	Female	38	35.0±9.8 (16–55)	65.3±3.2 (58.0–71.0)	65.3±3.2 (58.0–71.0)	−0.7±1.2 (−3.0–2.0)	21.13±1.92 (17.00–25.50)	5.16±1.31 (2.25–7.75)
	P value	-	0.720	0.002	0.001	0.255	0.267	0.416
Group M	Male	2	41.0±8.0 (33–49)	69.5±1.5 (68.0–71.0)	64.5±0.5 (64.0–65.0)	5.0±2.0 (3.0–7.0)	20.00±3.89 (17.25–22.75)	1.86±0.13 (1.75–2.00)
	Female	7	53.7±13.8 (31–76)	64.4±2.0 (62.0–68.0)	59.0±4.2 (52.0–66.0)	5.4±2.8 (2.0–10.0)	21.25±2.29 (18.5–25.00)	3.43±0.79 (2.25–5.00)
	P value	-	0.500	0.056	0.222	0.889	0.667	0.056
Group B	Male	3	38.3±4.0 (35–44)	73.7±3.9 (70.0–79.0)	72.7±4.1 (68.0–78.0)	1.0±0.8 (0–2.0)	26.00±0.87 (25.00–26.50)	5.00±0.71 (4.00–5.50)
	Female	19	38.4±11.9 (19–60)	65.0±2.2 (61.0–68.0)	63.1±3.0 (55.0–68.0)	1.9±2.3 (−3.0–6.0)	23.36±2.32 (19.00–27.50)	6.13±1.24 (3.00–8.25)
	P value	-	1.000	0.001	0.003	0.523	0.030	0.087

Statistical comparison using the Mann-Whitney *U* test.

**Table 4:** Gender-specific measurement results and statistical comparisons in controls.

	Number	Age (years)	Interpupillary distance (mm)	Exophthalmometry value (mm)
Male	59	36.3±10.1 (24–65)	64.7±3.5 (56.0–72.0)	14.05±2.15 (10.00–19.00)
Female	84	36.5±10.0 (24–57)	61.9±3.2 (55.0–69.0)	14.28±2.00 (9.25–19.00)
*P* value	-	0.934	< 0.001	0.520

Statistical comparison using Student's *t*-test.

Preoperative interpupillary distance in TED patients was significantly larger than that of controls (males, *P*<0.001; females, *P*<0.001). Preoperative exophthalmometry values in TED patients were higher than in controls (males, *P*<0.001; females, *P*<0.001).

**Figure 2:**
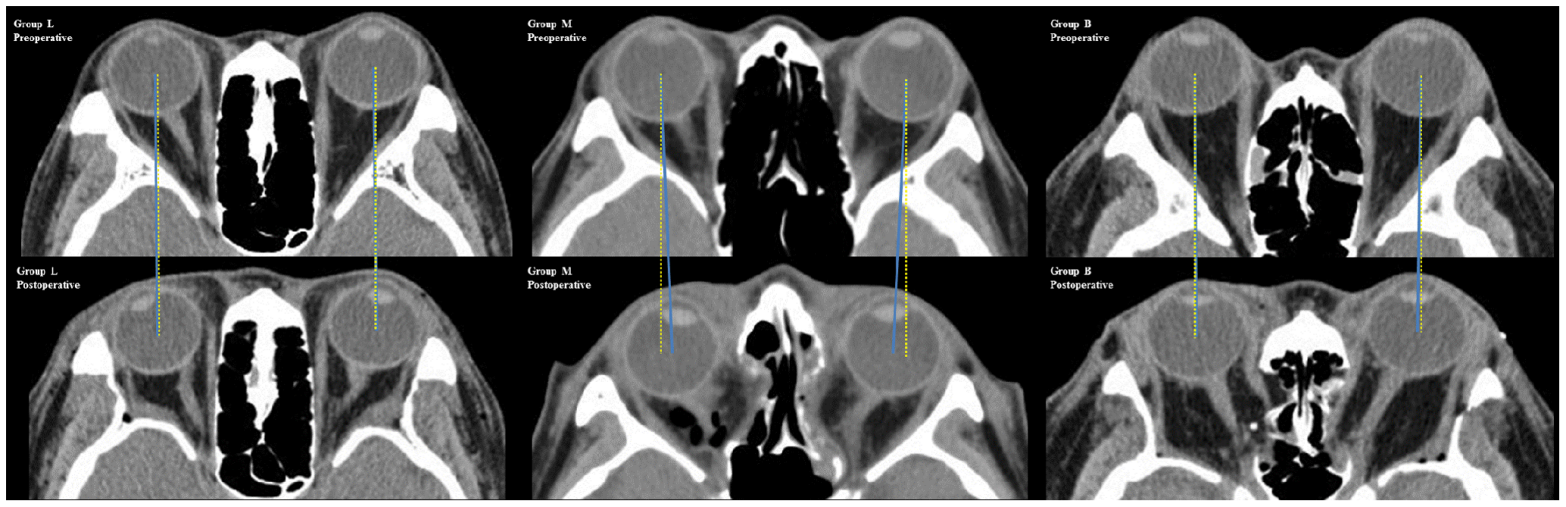
Axial computed tomographic (CT) images of the orbit before and after orbital decompression, in each group. The preoperative CT images have the same size as the postoperative images. The blue solid lines are connecting the center of the eyes, preoperatively and postoperatively, and the yellow dotted lines are perpendicular lines. In Group L (left), the eyes shifted slightly laterally after the lateral orbital wall decompression. In Group M (center), the eyes shifted medially after the medial orbital wall decompression. In Group B (right), the eyes shifted slightly medially after the balanced orbital decompression.

The interpupillary distance significantly increased postoperatively in Group L (*P* = 0.007) (Fig. 2). However, the distance was significantly reduced from the preoperative to the postoperative period in Groups M (*P*<0.001) and B (*P* = 0.029) (Fig. 2). The order of magnitude of the horizontal shift was Groups M>B>L (*P*<0.001). The proportion of sides with a positive horizontal shift were higher in Groups M and B than in Group L (*P*<0.001), although the proportion was not significantly different between Groups M and B (*P* = 0.101). Backward shifts of the eyes tended to be larger in Groups B, L, and M, in that order (Group L vs. M, *P* = 0.001; Group L vs. B, *P* = 0.065; Group M vs. B, *P*<0.001).

**Figure 3:**
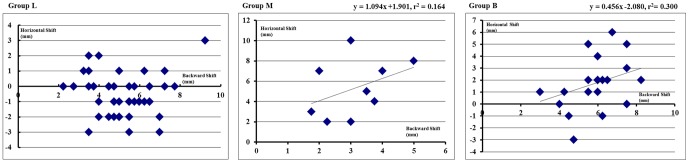
Scatter diagrams in each group with backward shift of the eyes on the x-axis and horizontal shift of the eyes on the y-axis.

There was little relationship between the horizontal and backward shifts in Group L (r = −0.03, *P* = 0.855) (Fig. 3). However, a moderate relationship between the horizontal and backward shifts was demonstrated in Groups M (y = 1.094× + 1.901; r = 0.405; r^2^ = 0.164; *P* = 0.280) and B (y = 0.456× − 2.080; r = 0.547; r^2^ = 0.300; *P* = 0.003) (Fig. 3), respectively.

The interpupillary distance was significantly larger in male than in female patients in all groups and in controls (*P*<0.050), except for in Group B (preoperative, *P* = 0.056; postoperative, *P* = 0.222). Horizontal eye shifts were not different between genders in each group (*P*>0.050).

## Discussion

The present study was the first to compare the interpupillary distance between TED patients and healthy volunteers, and to compare changes in the distances using different surgical procedures. A larger interpupillary distance was demonstrated in TED patients than in controls, preoperatively. However, the distance decreased after the medial orbital wall decompression and the balanced orbital decompression, but increased after the deep lateral orbital wall decompression. This study indicated that the medial orbital wall decompression or the balanced decompression can be used to correct both lateral and anterior displacement of the eyes in TED patients.

In the present study, the interpupillary distances were different among the subject groups. A principal causative factor of the difference was the anatomy of the bony orbit, which mainly determines the eye position [2], [16]. The lateral walls are oriented at an angle of 45 degrees lateral to the sagittal plane, whereas the medial walls of each orbit are oriented in the sagittal plane (Fig. 1) [17]. The eye position, therefore, shifts in the anterolateral or posteromedial direction along the lateral and medial orbital walls [2]. This could explain why proptotic TED patients showed laterally displaced eyes and why the eyes shifted medially after the medial orbital wall decompression. On the contrary, the lateral orbital wall decompression provided a posterolateral shift of the eyes, because the greater wing of the sphenoid is located posterior to the eye [13] and the lateral orbital rim, which is located lateral to the eye, was partially removed during the surgery (Fig. 1). As for the balanced decompression, the lateral shift by the lateral orbital wall decompression offset the medial shift by the medial orbital wall decompression.

Group B showed medial (1.8 mm) and posterior shifts (5.93 mm) with a linear correlation. This was somewhat different from the previous results of Alsuhaibani *et al.* [6], which demonstrated that the eyes shifted more medially (2.6 mm) and less posteriorly (2.5 mm) after balanced orbital decompression, although a linear correlation was not analyzed. A possible reason for this discrepancy was the different measurement methods of the horizontal and anteroposterior eye positions. In the previous study, the horizontal and anteroposterior eye positions were measured on axial and sagittal computed tomographic images [6], but were measured with autorefractor/keratometers and a Hertel exophthalmometer in the present study. Additionally, as the previous study was performed in the United States [6], a racial difference may be another influential factor for the different results.

Group L showed a mean lateral shift of 0.5 mm, which was smaller than that after lateral orbital wall decompression, as shown in the study by Fichter *et al*. (1.5 mm) [7]. Fichter *et al*. performed *en bloc* resection of the lateral orbital wall, including the whole lateral orbital rim [7], which may have caused a more lateral eye shift.

A gender-related difference was not observed in the horizontal and backward shifts of the eyes, in this study. Previous studies illustrated no remarkable gender-related differences in the anatomy of the lateral and medial orbital walls [18], [19], [20]. This indicated that the orbital space, newly obtained after medial or lateral orbital wall decompression, was not different between genders. This anatomical factor may be associated with non-gender related differences in the horizontal and backward shifts.

The interpupillary distance of controls in this study was somewhat shorter than that previously found in normal Japanese individuals aged 17–22 years (male, 66 mm; female, 64 mm) [21]. An increase in interpupillary distance with age, particularly until the second decade [22], [23], supported the difference in interpupillary distance. One of the possible reasons for the difference was a different environment. The previous study was performed over 40 years ago, in American-born individuals of Japanese ancestry in the United States [21]. Environment is relevant to body growth [24], affecting the interpupillary distance [4]. Use of calipers, in the previous study [21], may also be a reason for the different results, because the measurement values using calipers are influenced by strabismus.

Our study was limited by several factors. This study had a retrospective design and a small sample size in Group M. Another limitation was that this study was performed only based on a Japanese population. As some of the orbital anatomy exhibits racial differences [25], the results may not be applicable to other races.

In conclusion, this study demonstrated laterally shifted eyes in proptotic TED patients in comparison with normal individuals. However, the distance decreased after the medial orbital wall and the balanced orbital decompressions, but increased after lateral orbital wall decompressions. These results suggested that the medial orbital wall or the balanced decompression can be used to correct both lateral and anterior displacement of the eyes in TED patients.
